# The genetic architecture of susceptibility to parasites

**DOI:** 10.1186/1471-2148-8-187

**Published:** 2008-06-30

**Authors:** Lena Wilfert, Paul Schmid-Hempel

**Affiliations:** 1 Institute of Integrative Biology, ETH Zürich, ETH-Zentrum CHN, CH-8092 Zürich, Switzerland; 2 Institute of Evolutionary Biology, University of Edinburgh, King's Buildings, Edinburgh, EH9 3JT, UK

## Abstract

**Background:**

The antagonistic co-evolution of hosts and their parasites is considered to be a potential driving force in maintaining host genetic variation including sexual reproduction and recombination. The examination of this hypothesis calls for information about the genetic basis of host-parasite interactions – such as how many genes are involved, how big an effect these genes have and whether there is epistasis between loci. We here examine the genetic architecture of quantitative resistance in animal and plant hosts by concatenating published studies that have identified quantitative trait loci (QTL) for host resistance in animals and plants.

**Results:**

Collectively, these studies show that host resistance is affected by few loci. We particularly show that additional epistatic interactions, especially between loci on different chromosomes, explain a majority of the effects. Furthermore, we find that when experiments are repeated using different host or parasite genotypes under otherwise identical conditions, the underlying genetic architecture of host resistance can vary dramatically – that is, involves different QTLs and epistatic interactions. QTLs and epistatic loci vary much less when host and parasite types remain the same but experiments are repeated in different environments.

**Conclusion:**

This pattern of variability of the genetic architecture is predicted by strong interactions between genotypes and corroborates the prevalence of varying host-parasite combinations over varying environmental conditions. Moreover, epistasis is a major determinant of phenotypic variance for host resistance. Because epistasis seems to occur predominantly between, rather than within, chromosomes, segregation and chromosome number rather than recombination via cross-over should be the major elements affecting adaptive change in host resistance.

## Background

The interaction of hosts with their parasites is strongly affected by genotype [1] as shown, for example, by the interactions between host and parasite genotypes [2], the potential for increased parasite resistance by artificial selection [3], the direct identification of single genes [4], the molecular signature of selection in wild populations [5], and the improvement of resistance by genetic engineering [6]. Although these observations demonstrate the genetic basis of an interaction between host and parasite, typically they are silent about how the respective genes are arranged on the genome and whether and how they interact – they tell us little about the "genetic architecture" of loci involved in the interaction with parasites.

The genetic architecture of host resistance is closely linked to the evolution and ecological dynamics of host-parasite interactions. Sexual reproduction, for example, affects the combinations of genes that are involved in resistance to parasites in two different ways. First, pairs of genes located on the same chromosome are affected by meiotic recombination when crossing-over occurs between sister chromatids. This leads to an exchange of genetic material, such that new gene combinations are passed on to offspring. Second, pairs of genes located on different chromosomes are affected by segregation. In this process, sister chromosomes segregate and re-arrange themselves into new combinations in offspring. Whether or not such rearrangements in general improve or reduce the fitness of offspring, and whether a modifier coding for recombination and/or segregation can spread in a population, is a matter of ongoing debate (e.g. [7,8]). Furthermore, the *a priori *likelihood of any locus to be co-located with another relevant locus on the same chromosome depends on the organism's karyotype (the number of chromosomes). Hence, the genetic architecture of traits is of considerable interest as it affects host-parasite evolutionary interactions in many – albeit not fully understood – ways.

These questions call for a better knowledge of the genetic architecture of resistance, that is the number, effect size and interaction of loci determining resistance to parasites, as well as their genomic distribution. Today, this information can be provided by studies identifying the quantitative genetic basis of host resistance via a search for quantitative trait loci (QTL, table 1). In this process, a given "mapping population" (a set of individuals from defined genetic backgrounds, such as offspring of the same pedigree) is analysed such that the phenotype of interest (e.g. the level of resistance against a given infection) is measured for each individual. If the genotypes of these individuals are known, the phenotype can be statistically mapped onto a genetic linkage map. In this way, it is possible to locate "genes" of interest – QTLs representing small genomic regions – on a genetic map. QTLs are loci that account for a significant fraction of the phenotypic variation.

**Table 1:** Definition of technical terms as used in this study

additive QTL	Quantitative trait locus; a genetic locus whose alleles differentially affect a quantitative phenotype such as resistance to a parasite. The combined effect of additive QTL is equal to the sum of their individual effects. For the purpose of this study, we do not differentiate between purely additive, dominant and recessive QTL.
epistatic interaction	A non-additve interaction of genetic loci determining a phenotype, that is the combined effect of different alleles at these loci is different from the sum of the individual loci.
Interval mapping	Simple interval mapping tests whether an interval between two markers is significantly associated with a QTL.
Multiple-QTL-model mapping Composite-interval mapping	Both methods combine simple interval mapping with multiple regression. By thus controlling for the effects of other loci, these approaches allow for the accurate detection of multiple QTL defining a trait.

We have analysed the architecture of plant and animal parasite resistance as revealed in QTL studies. We use the identified QTLs to extract basic insights into how host resistance to parasites is genetically arranged on the genome. In a previous study, Kover and Caicedo [9] analysed QTLs for plant parasite resistance; they concluded that parasite resistance in plants is based on multiple loci, and found several cases where genetic interactions among loci – i.e. epistasis – substantially affected host resistance. In this study, we considerably expand the scope for fundamental questions about the genetic architecture of resistance by considering in detail the relevance of epistasis, as well as the persistence of the genetic architecture across different environmental factors and particular host-parasite associations.

## Results

Our study includes data from 194 publications (see Table 2, Table 3, and Additional file 1), representing a total of 500 QTL experiments. The studied host species cover a wide range of animals and plants. Among plants, the focus on crops reflects the importance of the genetic basis of resistance traits for use in marker-assisted breeding programs (Table 2). With the exception of one experiment [10], all experiments included a search for additive QTLs; epistatic interactions between these additive loci were examined in 86 experiments, with a further 62 cases in which the whole genome was scanned for epistatic interactions (i.e. in addition to the additive loci) (Table 3).

**Table 2:** Host-parasite associations included in study (total number of populations)^1^

Host	Virus	Bacteria	Protozoa	Fungi	Nematode
Animals					

*Aedes aegypti*	1 (4)	0	1 (4)	0	0
*Anopheles gambiae*	0	0	1 (5)	0	0
*Bombus terrestris*	0	0	1 (3)	0	0
*Bos taurus taurus *– cattle	1 (1) (prions)	0	1 (1)	0	0
*Gallus gallus *– chicken	1 (1)	1 (2)	0	0	0
*Mus musculus *– mouse	2 (5) (prions)	5 (14)	3 (7)	0	1 (1)
*Oncorhynchus mykiss*	1 (1)	0	0	0	0
*Rattus norvegicus *– rat	0	0	0	0	1 (1)
*Sus scrofa *– pig	1 (1)	0	0	0	0
*Tribolium castaneum*	0	0	0	0	1 (4)

Plants					

*Arabidopsis thaliana*	0	2 (2)	0	2 (4)	0
*Avena sativa *– oat	1 (2)	0	0	1 (5)	0
*Beta vulgaris *– sugar beet	0	0	0	1 (2)	0
*Brassica napus *– rapeseed	1 (1)	0	0	4 (10)	0
*Brassica oleracea *– cabbage etc.	0	1 (1)	0	0	0
*Brassica rapa *– cabbage etc.	0	0	0	1 (2)	0
*Capsicum annuum *– black pepper	1 (5)	0	0	1 (4)	0
*Cicer arietinum *– chickpea	0	0	0	1 (3)	0
*Cucumis melo *– melon	0	0	0	4 (20)	0
*Glycine sp. *– soybean	0	0	0	0	3 (4)
*Helianthus annuus *– sunflower	0	0	0	4 (15)	0
*Hevea brasiliensis *– rubber tree	0	0	0	1 (6)	0
*Hordeum chilense*	0	0	0	1 (1)	0
*Hordeum vulgare *– barley	1 (2)	0	0	13 (64)	0
*Lathyrus sativus *– grass pea	0	0	0	1 (1)	0
*Linum usitatissimum *– flax	0	0	0	1 (1)	0
*Lolium multiflorum *– Italian ryegrass	0	1 (2)	0	0	0
*Lolium perenne *– perennial ryegrass	0	0	0	1 (4)	0
*Lycopersicon spec. *– tomato	0	1 (1)	0	2 (3)	0
*Malus domesticus *– apple	0	1 (2)	0	2 (14)	0
*Manihot glaziovii *– ceara rubber tree	0	1 (16)	0	0	0
*Medicago sativa *– alfalfa	0	0	0	1 (1)	0
*Nicotiana sativum *– tobacco	0	1 (2)	0	0	0
*Oryza sativa *– rice	1 (3)	1 (2)	0	2 (18)	0
*Pennisetum glaucum *– pearl millet	0	0	0	1 (2)	0
*Phaseolus vulgaris *– bean	1 (1)	1 (4)	0	2 (5)	0
*Pisum sativum *– pea	0	0	0	3 (13)	0
*Populus deltoids *– poplar	0	0	0	1 (4)	0
*Prunus davidiana *– peach	1 (1)	0	0	1 (3)	0
*Rosa multiflora *– rose	0	0	0	1 (3)	0
*Solanum spec. *– potato	0	0	0	1 (5)	1 (1)
*Sorghum spec. *– sorghum	0	0	0	2 (12)	0
*Theobroma cacao *– cacao	0	0	0	4 (11)	0
*Triticum aestivum *– wheat	0	0	0	11 (102)	1 (2)
*Vicia faba *– fava bean	0	0	0	1 (3)	0
*Vigna radiata *– mung bean	0	0	0	1 (1)	0
*Zea mays *– maize	3 (12)	1 (2)	0	14 (36)	0

^1 ^List of references in additional files

**Table 3:** Number of studies used.

The study investigated...	Animals	Plants	Total cases
... only additive QTLs	36	316	352
... epistatic interactions of additive QTLs	10	76	86
... complete epistasis, and additive QTLs	9	53	62

Total studies	55	445	500

### Number of additive QTL and explained variance

**Figure 1:**
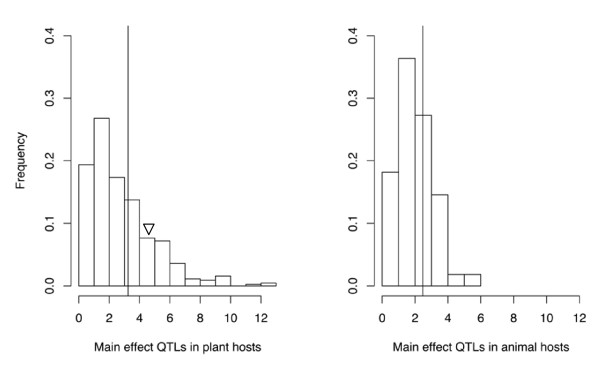
**Number of QTLs for resistance**. Number of additive loci reported for resistance/susceptibility in (left) plant and (right) animal hosts; a vertical line indicates the means (plants: 3.24 ± 2.22, *n *= 444; animals, 2.47 ± 1.18, *n *= 55). Triangle indicates the average value found in the study of Kover and Caicedo [9] (plants only).

We found that different studies revealed very different genetic architectures of host defence. The genetic basis of host resistance as described by additive QTLs (i.e. those representing the additive genetic effects) differs significantly between studies of animal (*n *= 55 populations) and plant hosts (*n *= 445 populations). Overall, additive QTLs were reported in 95.8% of all studies, that is, in 53 of 55 studies on animals and in 425 of 444 studies on plant hosts. The number of additive QTLs per mapping experiment ranged from 0 to 13 loci in plant hosts and 0 to 6 loci in animals. Accordingly, QTL studies of plant host resistance found significantly more QTLs on average than studies on animal hosts (number of QTLs: N_QTL_plants _= 3.24 ± 2.22 vs. N_QTL_animals _= 2.47 ± 1.18; Wilcoxon-test, *Z *= -1.973, *n *= 499, *P *< 0.05, Figure 1). Also, additive QTLs explained a higher proportion of the phenotypic variation for host resistance in plants than in animals (phenotypic variation explained: PVE_plants _= 48.66%, PVE_animals _= 33.03%; *Z *= -4.264, *n *= 444, *P *< 0.0001). To minimize the potential overestimation of phenotypic variation, this average is based on the total phenotypic variation explained by all significant QTL as determined by multiple regression where available; for those experiments in which only the PVE of individual QTLs is reported, we use the sum of all QTLs. Every individual QTL reported explained on average 16.7 ± 14.3% (*n *= 1'352) of phenotypic variation in plants and 13.6 ± 11.3% (*n *= 99) in animals, respectively (*Z *= -2.322, *n *= 1451, *P *< 0.05). Overall, the total explained variance increased with the number of additive loci reported (animal hosts: Spearman *r *= 0.5085, *P *< 0.001, *n *= 43; plant hosts: Spearman *r *= 0.3301, *P *< 0.0001, *n *= 411). In plants, each additional locus added less to the total (Explained variance per QTL: Spearman *r *= -0.61, *P *< 0.0001, *n *= 411), while we found no such relationship for animals (Spearman *r *= -0.0576, *P *= 0.581, *n *= 43). As a note of caution, the difference between plant and animal studies may be caused by differences in methodology, i.e. different crossing designs and the use of different statistical packages for estimating phenotypic variance. However, the difference in phenotypic variance appears to be robust across these criteria and these methodical aspects are thus unlikely to cloud the picture.

### Digenic epistatic effects

In the published studies, epistatic effects were analysed either between the previously identified additive QTLs only, or in the context of a whole genome scan. The latter therefore can uncover epistatic interactions between loci that otherwise did not rank as additive QTLs. Overall, for epistatic interactions, we found no difference between studies on animal and plant host resistance and therefore only report the pooled results. (i) Epistatic interactions between the additive QTLs were reported in 45.3% of cases (39 of 86 studies). A total of 28.5% of all additive QTLs were involved in epistatic interactions, and an average of 0.7 pair-wise interactions were found per study. (ii) With a genome-wide scan, epistatic interactions were found in 77.4% of studies (48 of 62 cases). An average of 1.9 pair-wise interactions was found per study and 25.3% of all additive QTLs were found to be involved in epistatic interactions. More importantly, 72.4% of epistatic loci had not been identified as additive QTLs (123 of 170 epistatic loci). In other words, considering only the additive QTLs (as is done in the first class of studies) massively underestimates (approximately by a factor of three) the number of epistatic interactions, as compared to whole genome scans. In fact, the extent of epistatic variance does not correlate with the amount of variance explained by the additive QTLs for either plants or animals ((Spearman *r *= 0.021, *P *= 0.82, *n *= 122). Note that from these studies, the sign of epistasis is difficult to assess because there is no *a priori *defined wildtype genotype.

### Co-location of loci on chromosomes or linkage groups

In many studies, at least two QTLs are co-located together on one chromosome or, respectively, one linkage group. In those studies – where more than one locus was reported analysing either only additive QTLs or additionally including epistatic interactions between these QTL – at least two co-located loci were found in 21.8% (n = 271 studies) and 37.2% (n = 78) of cases, respectively. In studies that included a genome-wide search for epistatic interactions, around half of the studies reported at least one pair of co-located loci (51.7%, *n *= 58). Of all loci mentioned in these studies, and combining additive QTLs and epistatic loci, a total of 22.0% are co-located with another locus on the same linkage group (*n *= 1'629 loci). However, an epistatic effect within the same linkage group was reported only in one study [11]. This study showed an interaction of two loci on linkage group three in two experiments on the infection severity of Cucumber mosaic virus on *Capsicum annuum *carried out at two different study sites. With the data set presented in this study, we could not determine whether the distribution of QTLs across linkage groups deviates from random, for example, whether QTLs would be clumped on some linkage groups rather than others. This is because there are typically either not enough QTLs in a given study population to be able to generate a statistically non-random distribution for the number of available linkage groups, or there are not enough linkage groups to accommodate the available QTLs in a way that can significantly deviate from random.

### Recovery of QTLs and epistatic interactions in different experiments

QTL studies often report the genetic basis of host resistance to a particular parasite under different conditions, i.e. when the experiment was repeated at different study sites, or involved different host or parasite genotypes. We asked whether additive QTLs or the pairs of loci involved in epistatic interactions identified for a certain host-parasite combination in one experiment are identical to those reported for another experiment within the same study. Typically experiments would differ in environmental condition or the particular host and parasite line used (from within the same host and parasite species). For example, many studies include several QTL experiments that – all else being equal – were carried out at different study sites or in different years. To ensure that the experiments were indeed comparable, we only include experiments carried out within the same study system, using identical phenotypic measurements and statistics.

We distinguish three categories of ways of repeating the QTL experiment under otherwise identical conditions: (1) Experiments conducted on identical pairs of host line and parasite isolate but in different years, environments or locations (factor "Environment" is varied). Furthermore, we distinguish between (2) experiments differing either in host line ("Host") or (3) parasite isolate ("Parasite"). For every comparison, we included only experiments that differed in a single factor; we further restricted the analysis to studies on plant hosts, since the available studies on animals were only ever repeated in different host genetic backgrounds but not years, environments or parasite lines. For each possible pair within a category we then checked for the recovery of the additive QTLs or epistatic loci.

**Figure 2:**
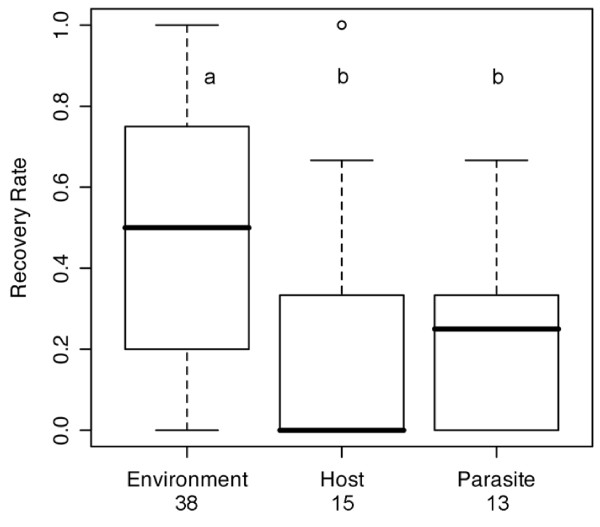
**Recovery rate of QTLs**. Box plot of recovery rate for QTL loci, i.e. what fraction of QTL loci remain the same between repeated studies. Recovery varies according to whether experiments are repeated in different environments (study site, year or environment, sample size indicated under x-Axis) or by using different host or parasite lines (*n *= 15; χ^2 ^= 15.79, *P *< 0.001). Letters indicate statistically similar groups in pairwise post-hoc tests (*t *= 1.968, α = 0.05). The horizontal line marks the median value, boxes indicate ± one quartile and vertical lines indicate the range of observations.

Comparing pairs of additive QTLs, recovery rate was significantly higher when the same host-parasite pair was tested in different environments than when different parasite isolates or host lines were used (Table 4, Figure 2). For studies including more than two repeated experiments, due to a limited sample size, we here considered all pairwise comparisons among experiments as independent of each other. With this proviso, we found a similar pattern as above in that differences in environmental factors were associated with higher recovery of loci (58.4%, *n *= 27) than differences in host (Host: 42.9%, *n *= 6) or parasite (28.7%, *n *= 115) lines used (χ^2 ^= 14.48, *P *< 0.001). While sample sizes were inevitably smaller for digenic epistatic interactions, recovery was again much higher for studies done in different environments (72.5%, *n *= 10) as compared to those using different parasite isolates (0%, *n *= 4) or host lines (0.09%, *n *= 1, χ^2 ^= 10.2, *P *< 0.01).

**Table 4:** Fraction of QTLs recovered in experiments repeated under various circumstances.

Factor	*n*	Fraction recovered	S.E.
Environmental variation	38	0.478	0.05
-- Different environments	5	0.490	0.14
-- Different years	23	0.489	0.06
-- Different locations	10	0.448	0.10
			
Different parasite isolates	13	0.246	0.08
Different host lines	15	0.128	0.08

The recovery rate varied for experiments using different lines of either host or parasite, or that were conducted under varying environmental conditions (χ^2 ^= 15.79, *P *< 0.001). Environmental variation included experiments conducted at multiple study sites, in different years or under different environmental conditions.

### Karyotype

The number of chromosomes (karyotype) varies considerably between species and sometimes between populations of the same species. Here, we assume that the karyotype sets a constraint for the observed variation in segregation and recombination, i.e. the karyotype is not itself selected for a given distribution of parasite-related genes.

We found that the karyotype did not correlate with the number of additive QTLs in either plants or animals (plants: Spearman's *r*_*s *_= 0.008, *P *= 0.87, *n *= 444; animals: *r*_*s *_= 0.18, *P *= 0.192, *n *= 55). However, overall, the number of epistatic interactions increased with the number of chromosomes (*r*_s _= 0.284, *P *= 0.0002, N = 147), and so did the number of digenic loci that were found (*r*_s _= 0.301, *P *= 0.0002, N = 147). When asking how many QTLs did co-locate on the same chromosome, we found that there was a significant negative correlation between the number of chromosomes and the number of co-located loci, as well as with the ratio of co-located loci to isolated loci per study (number of co-located loci: Spearman's *r*_s _= -0.119, *P *= 0.017; Ratio: *r*_s _= -0.150, *P *= 0.003, *N *= 404).

## Discussion

The large number of studies that have used the QTL technique to identify and locate genes or small genetic regions that explain observed variation in resistance to parasites offers a window into the genetic architecture of resistance traits. The available studies cover a wide range of taxa, from plants to protozoa, fungi, and to animals. Even though a total of *n *= 500 studies were included here, no attempt was made to account for phylogenetic dependencies, since for any one taxon the number of studies would be too small and the relevant large-scale phylogeny has often not been sufficiently ascertained. Instead, we consider our results as providing a snapshot into the genetic architecture of disease resistance – such as how many loci are involved, where are they located and whether there are any interactions.

Our results are in broad accordance with a previous study on plant QTLs [9]. We found that resistance is typically based on a limited number of loci (Fig. 1) and that additive QTLs explain a considerable proportion of the observed phenotypic variation. While this is a robust finding, one needs to bear in mind that QTL studies are inherently biased towards an underestimation of the number of involved loci, while overestimating the effect of identified QTLs [12]. It is interesting to note that studies on animal host resistance generally found fewer additive QTLs, which explained less of the total phenotypic variance, than studies on plant resistance. No such difference was found for the epistatic interactions. It should be noted that this difference might be caused by methodological differences. Whereas a limited sample size is frequently a concern in animal studies, studying plant cultivars offers the opportunity of creating very large datasets from controlled crosses. Thus, the power to detect QTL may potentially be somewhat higher in the available studies on plant hosts. In addition, there is some variation in the QTL mapping algorithms used in different studies. A large proportion of plant studies use composite-interval-mapping [13], whereas multiple-QTL-mapping [14] is comparatively more common in the animal studies analysed here. While both methods rely on combining simple interval methods with multiple regression (see table 1), their implementation in software packages is not identical, which can lead to slightly different results in practice (e.g. [15]). In spite of this pattern, there are no clear-cut methodological differences between studies on animal and plant hosts. Thus, our results raise the possibility that the genetic architecture of resistance may indeed differ between animals and plants. For example, animal resistance may involve many loci of small effect that remained undetected in these studies.

Our analysis shows that epistatic interactions are a major component of the genetic architecture of parasite resistance. Across studies, the variance explained by epistasis is not correlated to the additive variance. Epistatic interactions add a substantial, if not a major, amount of explained variance. Hence, we conclude that by ignoring epistatic interactions, the quantitative genetic basis of resistance would be massively underestimated. Similarly, there is in general little overlap between the QTLs identified as additive loci and those associated with epistatic interactions. Even though this difference may be partly due to the method of QTL-identification, the findings collectively suggest that disease resistance is strongly affected by epistatically interacting loci that are not additive loci.

With respect to the location of the QTLs, we find that at least two QTLs are regularly located on the same chromosome or linkage group. However, typically, these loci are additive QTLs, while only in one of 148 experiments an epistatic interaction within a linkage group was reported. It must be noted that there is a bias against discovering epistatic interactions between closely linked loci due to the limited statistical power of the method. Nevertheless, the data suggest that epistatic interactions occur to an important extent between loci on different linkage groups or chromosomes. Furthermore, for some of the species in which epistatic interactions have been reported (*Avena sativa, Brassica napus, Capsicum annuum, Cucumis melo, Linum usitatissimum *and *Bombus terrestris*) some of the currently described linkage groups will eventually be merged into a single chromosome as the resolution of the linkage map improves. But given the strong tendency for between-rather than within-chromosome epistasis, these reservations are unlikely to substantially change the above conclusion. Hence, we suggest that epistatic interactions will mostly be affected by segregation of chromosomes rather than by within-chromosome recombination. As a consequence, karyotype will play an important role for the effect of these two different processes on the combination of QTLs in offspring. Indeed, the number of epistatic interactions increases with the number of chromosomes.

Finally, we also find that the identity of the involved QTLs is unstable. In those cases, where the same organism was studied repeatedly, either with different host-parasite line combinations, or in different years, environments or locations, the recovery rate of the QTLs was very poor. In fact, as a rule, different QTLs show up in different experiments even though they all pertain to resistance against the same parasite species. This phenomenon of "shifting loci" is well known from plant breeding, where it is an obstacle to marker-assisted breeding efforts [16]. Shifting loci also add to the variance in epistatic interactions. Shifting epistatic interactions can also be found experimentally; a recent study by Pepin and Wichman [17] on host recognition sites in a bacteriophage showed that there is not only variation in the involved loci but also in the sign of epistasis in different environments and when interacting with different host genotypes. At least in theory, such variance in epistatic interactions reduces the effects of selection for increased recombination [18]. But as Kouyos et al. [19] have shown, even few interactions with weak effect as observed in the studies we surveyed may drive recombination, rather than the average epistasis of all interactions.

## Conclusion

We found that loci shift more – that is, loci can be recovered less frequently – when the particular combination of the host-parasite lines is changed (i.e. the respective genetic background changes) as compared to the higher recovery when the environment is changed (for the identical host-parasite combination). As far as the genetic architecture of quantitative variation in resistance is concerned, this difference shows that environmental variation may not always override the effect of variation in parasites [20]. Indeed, it corroborates the importance of genotypic interactions in host-parasite systems.

Our evaluation of published QTL-studies suggests that the genetic architecture of parasite resistance gives epistasis an important role, especially where it occurs between chromosomes, and that these effects are highly variable across different host-parasite pairings. Furthermore, these epistatic interactions are primarily affected by segregation and chromosome number, rather than by recombination via cross-over within a chromosome. Such constraints set by the genetic architecture of parasite resistance should have major effects on the evolution and maintenance of segregation (i.e. of sex per se) and within-chromosome recombination.

## Methods

Using the Web of Science [21], we screened the published literature for studies that have taken a QTL approach to reveal the genetic basis of host resistance or susceptibility. To maximise consistency and comparability in the extracted data, we include only studies that fulfilled the two following criteria. (1) Studies had to employ interval mapping or similar methods, e.g. composite interval mapping or multiple-QTL-model mapping, resulting in precise information on the number and location of QTLs. Studies using less precise non-parametric or not generally recognized genetic mapping approaches were excluded to maximise comparability. (2) The screen for QTLs had to encompass the entire available genome, and not limit itself to certain candidate genes or genetic areas of interest.

Many studies investigate the genetic architecture of host defence using several independent experiments or for several traits. If a study reports the genetic architecture of a trait measured under different environmental conditions (e.g. if the host-parasite interaction was assayed in different years or locations) or with different combinations of host or parasite lines, the results of each experiment were included as independent data points in the present dataset. Frequently, several traits were measured in the same experiment, for example disease severity in several tissues. In this situation, we included only one trait per experiment, by choosing prior to the analyses the trait that is expected to be most relevant in a host-parasite co-evolution scenario (for example parasite load would be more informative than secondary disease symptoms, and survival time after infection would be more important than measures of the magnitude of the immune response).

The data compiled for this study consists of count data with a limited range (number of QTLs, epistatic interactions and chromosomes) or percentages (explained phenotypic variation, recovery rate of loci between experiments). To accommodate this data structure, we have used standard non-parametric tests throughout the analyses.

## Authors' contributions

LW extracted data from published QTL studies. Both LW and PSH contributed to the design of this study, the statistical analyses and the writing of the manuscript.

## Supplementary Material

Additional file 1 **Table A1: References for data on the genetic architecture of host-parasite interactions**. Table and bibliography of all studies included in the analyses of genetic architecture. Click here for file
