# Supplementary material for: The genetic architecture of susceptibility to parasites
Source: BMC Evol Biol. 2008 Jun 30;8:187. doi: 10.1186/1471-2148-8-187 (PMC2446395; doi:10.1186/1471-2148-8-187)
Supplement: Additional file 1 — Table A1: References for data on the genetic architecture of host-parasite interactions. Table and bibliography of all studies included in the analyses of genetic architecture. [file 1471-2148-8-187-S1.pdf]

Table A1: References for data on the genetic architecture of host-parasite interactions

	Virus	Bacteria	Protozoa	Fungi	Nematode
Animal					
Aedes aegypti	[1-3]		[4, 5]		
Anopheles gambiae			[6]		
Bombus terrestris			[7]		
Bos taurus	[8]		[9]		
Gallus gallus	[10]	[11]			
Mus musculus	[12-15]	[16-24]	[25-29]		[30]
Oncorhynchus mykiss	[31]				
Rattus norvegicus					[32]
Sus scrofa	[33]				
Tribolium castaneum					[34, 35]
Plants	Virus	Bacteria	Protozoa	Fungi	Nematode
Arabidopsis thaliana		[36, 37]		[38-40]	
Avena sativa	[41]			[42, 43]	
Beta vulgaris				[44, 45]	
Brassica napus	[46]			[47-51]	
Brassica oleracea		[52]			
Brassica rapa				[53]	
Capsicum annuum	[54, 55]			[56]	
Cicer arietinum				[57, 58]	
Cucumis melo				[59, 60]	
Glycine spec					[61-63]
Helianthus annuus				[64-70]	
Hevea brasiliensis				[71, 72]	
Hordeum chilense				[73]	
Hordeum vulgare	[74]			[75-92]	
Lathyrus sativus				[93]	
Linum usitassimum				[94]	
Lolium multiflorum		[95]			
Lolium perenne				[96]	
Lycopersicon spec.		[97]		[98, 99]	
Malus domesticus		[100]		[101-103]	
Manihot glaziovii		[104]			
Medicago sativa				[105]	
Nicotiana sativum		[106]			

	Virus	Bacteria	Protozoa	Fungi	Nematode
Oryza sativa	[107, 108]	[109, 110]		[111-118]	
Pennisetum glaucum				[119]	
Phaseolus vulgaris	[120]	[120-122]		[120, 123, 124]	
Pisum sativum				[125-128]	
Populus deltoides				[129]	
Prunus davidiana	[130]			[131]	
Rosa multiflora				[132]	
Solanum spec.				[133-135]	[136]
Sorghum spec.				[137, 138]	
Theobroma cacao				[139-142]	
Triticum aestivum				[143-169]	[170]
Vicia faba				[171, 172]	
Vigna radiata				[173]	
Zea mays	[174-179]	[180]		[180-194]	

References

1. Gomez-Machorro C, Bennett KE, Munoz MD, Black WC: **Quantitative trait loci affecting dengue midgut infection barriers in an advanced intercross line of *Aedes aegypti***. *Ins Mol Biol* 2004, **13**(6):637-648.
2. Bosio CF, Fulton RE, Salasek ML, Beaty BJ, Black WC: **Quantitative trait loci that control vector competence for dengue-2 virus in the mosquito *Aedes aegypti***. *Genetics* 2000, **156**(2):687-698.
3. Bennett KE, Flick D, Fleming KH, Jochim R, Beaty BJ, Black WC: **Quantitative trait loci that control dengue-2 virus dissemination in the mosquito *Aedes aegypti***. *Genetics* 2005, **170**(1):185-194.
4. Severson DW, Thathy V, Mori A, Zhang Y, Christensen BM: **Restriction-Fragment-Length-Polymorphism Mapping of Quantitative Trait Loci for Malaria Parasite Susceptibility in the Mosquito *Aedes aegypti***. *Genetics* 1995, **139**(4):1711-1717.
5. Zhong DB, Menge DM, Temu EA, Chen H, Yan GY: **Amplified fragment length polymorphism mapping of quantitative trait loci for malaria parasite susceptibility in the yellow fever mosquito Aedes aegypti**. *Genetics* 2006, **173**(3):1337-1345.
6. Menge DM, Zhong DB, Guda T, Gouagna L, Githure J, Beier J, Yan GY: **Quantitative trait loci controlling refractoriness to *Plasmodium falciparum* in natural *Anopheles gambiae* mosquitoes from a malaria-endemic region in western Kenya**. *Genetics* 2006, **173**(1):235-241.
7. Wilfert L, Gadau J, Baer B, Schmid-Hempel P: **Natural variation in the genetic architecture of a host-parasite interaction in the bumblebee *Bombus terrestris***. 2007, **16**(6):1327-1339.
8. Zhang C, de Koning DJ, Hernandez-Sanchez J, Haley CS, Williams JL, Wiener P: **Mapping of multiple quantitative trait loci affecting bovine spongiform encephalopathy**. *Genetics* 2004, **167**(4):1863-1872.
9. Hanotte O, Ronin Y, Agaba M, Nilsson P, Gelhaus A, Horstmann R, Sugimoto Y, Kemp S, Gibson J, Korol A, Soller M, Teale A: **Mapping of quantitative trait loci controlling trypanotolerance in a cross of tolerant West African N'Dama and susceptible East African Boran cattle**. *Proc Natl Acad Sci USA* 2003, **100**(13):7443-7448.
10. Yonash N, Bacon LD, Witter RL, Cheng HH: **High resolution mapping and identification of new quantitative trait loci (QTL) affecting susceptibility to Marek's disease**. *Anim Genet* 1999, **30**(2):126-135.
11. Tilquin P, Barrow PA, Marly J, Pitel F, Plisson-Petit F, Velge P, Vignal A, Baret PV, Bumstead N, Beaumont C: **A genome scan for quantitative trait loci affecting the Salmonella carrier-state in the chicken**. *Genet Select Evol* 2005, **37**(5):539-561.
12. Moreno CR, Lantier F, Lantier I, Sarradin P, Elsen JM: **Detection of new quantitative trait loci for susceptibility to transmissible spongiform encephalopathies in mice**. *Genetics* 2003, **165**(4):2085-2091.
13. Lloyd SE, Onwuazor ON, Beck JA, Mallinson G, Farrall M, Targonski P, Collinge J, Fisher EMC: **Identification of multiple quantitative trait loci linked to prion disease incubation period in mice**. *Proc Natl Acad Sci U S A* 2001, **98**(11):6279-6283.

14. Manolakou K, Beaton J, McConnell I, Farquar C, Manson J, Hastie ND, Bruce M, Jackson IJ: **Genetic and environmental factors modify bovine spongiform encephalopathy incubation period in mice**. *Proc Natl Acad Sci U S A* 2001, **98**(13):7402-7407.
15. Stephenson DA, Chiotti K, Ebeling C, Groth D, DeArmond SJ, Prusiner SB, Carlson GA: **Quantitative trait loci affecting prion incubation time in mice**. *Genomics* 2000, **69**(1):47-53.
16. Roper RJ, Weis JJ, McCracken BA, Green CB, Ma Y, Weber KS, Fairbairn D, Butterfield RJ, Potter MR, Zachary JF, Doerge RW, Teuscher C: **Genetic control of susceptibility to experimental Lyme arthritis is polygenic and exhibits consistent linkage to multiple loci on chromosome 5 in four independent mouse crosses**. *Genes Immun* 2001, **2**(7):388-397.
17. Weis JJ, McCracken BA, Ma Y, Fairbairn D, Roper RJ, Morrison TB, Weis JH, Zachary JF, Doerge RW, Teuscher C: **Identification of quantitative trait loci governing arthritis severity and humoral responses in the murine model of Lyme disease**. *J Immunol* 1999, **162**(2):948-956.
18. Bernstein-Hanley I, Balsara ZR, Ulmer W, Coers J, Starnbach MN, Dietrich WF: **Genetic analysis of susceptibility to *Chlamydia trachomatis* in mouse**. *Genes Immun* 2006, **7**(2):122-129.
19. Lavebratt C, Apt AS, Nikonenko BV, Schalling M, Schurr E: **Severity of tuberculosis in mice is linked to distal chromosome 3 and proximal chromosome 9**. *J Infect Dis* 1999, **180**(1):150-155.
20. Mitsos LM, Cardon LR, Fortin A, Ryan L, LaCourse R, North RJ, Gros P: **Genetic control of susceptibility to infection with *Mycobacterium tuberculosis* in mice**. *Genes Immun* 2000, **1**(8):467-477.
21. Yan BS, Kirby A, Shebzukhov YV, Daly MJ, Kramnik I: **Genetic architecture of tuberculosis resistance in a mouse model of infection**. *Genes Immun* 2006, **7**(3):201-210.
22. Caron J, Loredo-Osti JC, Laroche L, Skamene E, Morgan K, Malo D: **Identification of genetic loci controlling bacterial clearance in experimental *Salmonella enteritidis* infection: an unexpected role of Nramp1 (Slc11a1) in the persistence of in infection in mice**. *Genes Immun* 2002, **3**(4):196-204.
23. Sebastiani G, Olien L, Gauthier S, Skamene E, Morgan K, Gros P, Malo D: **Mapping of genetic modulators of natural resistance to infection with *Salmonella typhimurium* in wild-derived mice**. *Genomics* 1998, **47**(2):180-186.
24. de Souza CM, Morel L, Cabrera WHK, Starobinas N, Ribeiro OG, Siqueira M, Ibanez OM, De Franco M: **Quantitative trait loci in chromosomes 3, 8, and 9 regulate antibody production against Salmonella flagellar antigens in the mouse**. 2004, **15**(8):630-636.
25. Roberts LJ, Baldwin TM, Curtis JM, Handman E, Foote SJ: **Resistance to *Leishmania major* is linked to the H2 region on chromosome 17 and to chromosome 9**. *J Exp Med* 1997, **185**(9):1705-1710.
26. Burt RA, Baldwin TM, Marshall VM, Foote SJ: **Temporal expression of an H2-linked locus in host response to mouse malaria**. *Immunogenetics* 1999, **50**(5-6):278-285.

27. Foote SJ, Burt RA, Baldwin TM, Presente A, Roberts AW, Laural YL, Lew AM, Marshall VM: **Mouse loci for malaria-induced mortality and the control of parasitaemia**. *Nat Genet* 1997, **17**(4):380-381.
28. Fortin A, Belouchi A, Tam MF, Cardon L, Skamene E, Stevenson MM, Gros P: **Genetic control of blood parasitaemia in mouse malaria maps to chromosome 8**. *Nat Genet* 1997, **17**(4):382-383.
29. Kemp SJ, Iraqi F, Darvasi A, Soller M, Teale AJ: **Localization of genes controlling resistance to trypanosomiasis in mice**. *Nature Genet* 1997, **16**(2):194-196.
30. Iraqi FA, Behnke JM, Menge DM, Lowe A, Teale AJ, Gibson JP, Baker LR, Wakelin D: **Chromosomal regions controlling resistance to gastro-intestinal nematode infections in mice**. *Mamm Genome* 2003, **14**(3):184-191.
31. Ozaki A, Sakamoto T, Khoo S, Nakamura K, Coimbra MRM, Akutsu T, Okamoto N: **Quantitative trait loci (QTLs) associated with resistance/susceptibility to infectious pancreatic necrosis virus (IPNV) in rainbow trout (*Oncorhynchus mykiss*)**. *Mol Genet Genomics* 2001, **265**(1):23-31.
32. Suzuki T, Ishih A, Kino H, Muregi FW, Takabayashi S, Nishikawa T, Takagi H, Terada M: **Chromosomal mapping of host resistance loci to *Trichinella spiralis* nematode infection in rats**. *Immunogenetics* 2006, **58**(1):26-30.
33. Reiner G, Melchinger E, Kramarova M, Pfaff E, Buttner M, Saalmuller A, Geldermann H: **Detection of quantitative trait loci for resistance/susceptibility to pseuclorabies virus in swine**. *J Gen Virol* 2002, **83**:167-172.
34. Zhong DB, Pai A, Yan GY: **Quantitative trait loci for susceptibility to tapeworm infection in the red flour beetle**. *Genetics* 2003, **165**(3):1307-1315.
35. Zhong DB, Pai A, Yan GY: **Costly resistance to parasitism: Evidence from simultaneous quantitative rait loci mapping for resistance and fitness in *Tribolium castaneum***. *Genetics* 2005, **169**(4):2127-2135.
36. Buell CR, Somerville SC: **Use of *Arabidopsis* recombinant inbred lines reveals a monogenic and a novel digenic resistance mechanism to *Xanthomonas campestris* pv *campestris***. *Plant J* 1997, **12**(1):21-29.
37. Kover PX, Wolf JB, Kunkel BN, Cheverud JM: **Genetic architecture of *Arabidopsis thaliana* response to infection by *Pseudomonas syringae***. *Heredity* 2005, **94**(5):507-517.
38. Denby KJ, Kumar P, Kliebenstein DJ: **Identification of *Botrytis cinerea* susceptibility loci in *Arabidopsis thaliana***. *Plant J* 2004, **38**(3):473-486.
39. Schiff CL, Wilson IW, Somerville SC: **Polygenic powdery mildew disease resistance in *Arabidopsis thaliana*: quantitative trait analysis of the accession Warschau-1**. *Plant Pathol* 2001, **50**(6):690-701.
40. Wilson IW, Schiff CL, Hughes DE, Somerville SC: **Quantitative trait loci analysis of powdery mildew disease resistance in the *Arabidopsis thaliana* accession Kashmir-1**. *Genetics* 2001, **158**(3):1301-1309.
41. Zhu S, Kolb FL, Kaeppler HF: **Molecular mapping of genomic regions underlying barley yellow dwarf tolerance in cultivated oat (*Avena sativa* L.)**. *Theor Appl Gen* 2003, **106**(7):1300-1306.
42. Zhu SQ, Leonard KJ, Kaeppler HF: **Quantitative trait loci associated with seedling resistance to isolates of *Puccinia coronata* in oat**. *Phytopathology* 2003, **93**(7):860-866.

43. Zhu S, Kaeppler HF: **Identification of quantitative trait loci for resistance to crown rust in oat line MAM17-5**. *Crop Sci* 2003, **43**(1):358-366.
44. Nilsson NO, Hansen M, Panagopoulos AH, Tuvesson S, Ehlde M, Christiansson M, Rading IM, Rissler M, Kraft T: **QTL analysis of Cercospora leaf spot resistance in sugar beet**. *Plant Breed* 1999, **118**(4):327-334.
45. Setiawan A, Koch G, Barnes SR, Jung C: **Mapping quantitative trait loci (QTLs) for resistance to Cercospora leaf spot disease (*Cercospora beticola* Sacc.) in sugar beet (*Beta vulgaris* L.)**. *Theor Appl Gen* 2000, **100**(8):1176-1182.
46. Dreyer F, Graichen K, Jung C: **A major quantitative trait locus for resistance to Turnip Yellows Virus (TuYV, syn. beet western yellows virus, BWYV) in rapeseed**. *Plant Breed* 2001, **120**(6):457-462.
47. Manzanares-Dauleux MJ, Delourme R, Baron F, Thomas G: **Mapping of one major gene and of QTLs involved in resistance to clubroot in *Brassica napus***. *Theor Appl Gen* 2000, **101**(5-6):885-891.
48. Pilet ML, Duplan G, Archipiano H, Barret P, Baron C, Horvais R, Tanguy X, Lucas MO, Renard M, Delourme R: **Stability of QTL for field resistance to blackleg across two genetic backgrounds in oilseed rape**. *Crop Sci* 2001, **41**(1):197-205.
49. Zhao JW, Meng JL: **Genetic analysis of loci associated with partial resistance to *Sclerotinia sclerotiorum* in rapeseed (*Brassica napus* L.)**. *Theor Appl Gen* 2003, **106**(4):759-764.
50. Pilet ML, Delourme R, Foisset N, Renard R: **Identification of loci contributing to quantitative field resistance to blackleg disease, causal agent *Leptosphaeria maculans* (Desm.) Ces. et de Not., in Winter rapeseed (*Brassica napus* L.)**. *Theor Appl Gen* 1998, **96**(1):23-30.
51. Pilet ML, Delourme R, Foisset N, Renard M: **Identification of QTL involved in field resistance to light leaf spot (*Pyrenopeziza brassicae*) and blackleg resistance (*Leptosphaeria maculans*) in winter rapeseed (*Brassica napus* L.)**. *Theor Appl Gen* 1998, **97**(3):398-406.
52. Camargo LEA, Williams PH, Osborn TC: **Mapping of Quantitative Trait Loci Controlling Resistance of *Brassica oleracea* to *Xanthomonas campestris* pv *campestris* in the Field and Greenhouse**. *Phytopathology* 1995, **85**(10):1296-1300.
53. Kole C, Williams PH, Rimmer SR, Osborn TC: **Linkage mapping of genes controlling resistance to white rust (*Albugo candida*) in *Brassica rapa* (syn. *campestris*) and comparative mapping to *Brassica napus* and *Arabidopsis thaliana***. *Genome* 2002, **45**(1):22-27.
54. Ben Chaim A, Grube RC, Lapidot M, Jahn M, Paran I: **Identification of quantitative trait loci associated with resistance to cucumber mosaic virus in *Capsicum annuum***. *Theor Appl Genet* 2001, **102**(8):1213-1220.
55. Caranta C, Pflieger S, Lefebvre V, Daubeze AM, Thabuis A, Palloix A: **QTLs involved in the restriction of cucumber mosaic virus (CMV) long-distance movement in pepper**. *Theor Appl Genet* 2002, **104**(4):586-591.
56. Lefebvre V, Daubeze AM, van der Voort JR, Peleman J, Bardin M, Palloix A: **QTLs for resistance to powdery mildew in pepper under natural and artificial infections**. *Theor Appl Gen* 2003, **107**(4):661-666.
57. Flandez-Galvez H, Ades PK, Ford R, Pang ECK, Taylor PWJ: **QTL analysis for ascochyta blight resistance in an intraspecific population of chickpea (*Cicer arietinum* L.)**. *Theor Appl Genet* 2003, **107**(7):1257-1265.

58. Santra DK, Tekeoglu M, Ratnaparkhe M, Kaiser WJ, Muehlbauer FJ: **Identification and mapping of QTLs conferring resistance to ascochyta blight in chickpea**. *Crop Sci* 2000, **40**(6):1606-1612.
59. Perchepied L, Bardin M, Dogimont C, Pitrat A: **Relationship between loci conferring downy mildew and powdery mildew resistance in melon assessed by quantitative trait loci mapping**. *Phytopathology* 2005, **95**(5):556-565.
60. Perchepied L, Dogimont C, Pitrat M: **Strain-specific and recessive QTLs involved in the control of partial resistance to *Fusarium oxysporum* f. sp *melonis* race 1.2 in a recombinant inbred line population of melon**. *Theor Appl Gen* 2005, **111**(1):65-74.
61. Wang D, Arelli PR, Shoemaker RC, Diers BW: **Loci underlying resistance to Race 3 of soybean cyst nematode in *Glycine soja* plant introduction 468916**. *Theor Appl Gen* 2001, **103**(4):561-566.
62. Tamulonis JP, Luzzi BM, Hussey RS, Parrott WA, Boerma HR: **RFLP mapping of resistance to southern root-knot nematode in soybean**. *Crop Sci* 1997, **37**(6):1903-1909.
63. Tamulonis JP, Luzzi BM, Hussey RS, Parrott WA, Boerma HR: **DNA marker analysis of loci conferring resistance to peanut root-knot nematode in soybean**. *Theor Appl Gen* 1997, **95**(4):664-670.
64. Bert PF, Jouan I, de Labrouhe DT, Serre F, Nicolas P, Vear F: **Comparative genetic analysis of quantitative traits in sunflower (*Helianthus annuus* L.) 1. QTL involved in resistance to *Sclerotinia sclerotiorum* and *Diaporthe helianthi***. *Theor Appl Genet* 2002, **105**(6-7):985-993.
65. Al-Chaarani G, Roustaee A, Gentzbittel L, Mokrani L, Barrault G, Dechamp-Guillaume G, Sarrafi A: **A QTL analysis of sunflower partial resistance to downy mildew (*Plasmopara halstedii*) and black stem (*Phoma macdonaldii*) by the use of recombinant inbred lines (RILs)**. *Theor Appl Genet* 2002, **104**(2-3):490-496.
66. Bert PF, Dechamp-Guillaume G, Serre F, Jouan I, de Labrouhe DT, Nicolas P, Vear F: **Comparative genetic analysis of quantitative traits in sunflower (*Helianthus annuus* L.) - 3. Characterisation of QTL involved in resistance to *Sclerotinia sclerotiorum* and *Phoma macdonaldi***. *Theor Appl Genet* 2004, **109**(4):865-874.
67. Mestries E, Gentzbittel L, de Labrouhe DT, Nicolas P, Vear F: **Analyses of quantitative trait loci associated with resistance to *Sclerotinia sclerotiorum* in sunflowers (*Helianthus annuus* L) using molecular markers**. *Mol Breed* 1998, **4**(3):215-226.
68. Ronicke S, Hahn V, Vogler A, Friedt W: **Quantitative trait loci analysis of resistance to *Sclerotinia sclerotiorum* in sunflower**. *Phytopathology* 2005, **95**(7):834-839.
69. Micic Z, Hahn V, Bauer E, Schon CC, Knapp SJ, Tang S, Melchinger AE: **QTL mapping of Sclerotinia midstalk-rot resistance in sunflower**. *Theor Appl Gen* 2004, **109**(7):1474-1484.
70. Micic Z, Hahn V, Bauer E, Melchinger AE, Knapp SJ, Tang S, Schon CC: **Identification and validation of QTL for Sclerotinia midstalk rot resistance in sunflower by selective genotyping**. *Theor Appl Gen* 2005, **111**(2):233-242.
71. Lespinasse D, Grivet L, Troispoux V, Rodier-Goud M, Pinard F, Seguin M: **Identification of QTLs involved in the resistance to South American leaf blight (*Microcyclus ulei*) in the rubber tree**. *Theor Appl Gen* 2000, **100**(6):975-984.

72. Le Guen V, Lespinasse D, Oliver G, Rodier-Goud M, Pinard F, Seguin M: **Molecular mapping of genes conferring field resistance to South American Leaf Blight (*Microcyclus ulei*) in rubber tree**. *Theor Appl Gen* 2003, **108**(1):160-167.
73. Patto MCV, Rubiales D, Martin A, Hernandez P, Lindhout P, Niks RE, Stam P: **QTL mapping provides evidence for lack of association of the avoidance of leaf rust in *Hordeum chilense* with stomata density**. *Theor Appl Gen* 2003, **106**(7):1283-1292.
74. Scheurer KS, Friedt W, Huth W, Waugh R, Ordon F: **QTL analysis of tolerance to a German strain of BYDV-PAV in barley (*Hordeum vulgare* L.)**. *Theor Appl Gen* 2001, **103**(6-7):1074-1083.
75. Emebiri LC, Platz G, Moody DB: **Disease resistance genes in a doubled haploid population of two-rowed barley segregating for malting quality attributes**. *Aust J Agric Res* 2005, **56**(1):49-56.
76. Richter K, Schondelmaier J, Jung C: **Mapping of quantitative trait loci affecting *Drechslera teres* resistance in barley with molecular markers**. *Theor Appl Gen* 1998, **97**(8):1225-1234.
77. Read BJ, Raman H, McMichael G, Chalmers KJ, Ablett GA, Platz GJ, Raman R, Genger RK, Boyd WJR, Li CD, Grime CR, Park RF, Wallwork H, Prangnell R, Lance RCM: **Mapping and QTL analysis of the barley population Sloop x Halcyon**. *Aust J Agric Res* 2003, **54**(11-12):1145-1153.
78. Dahleen LS, Agrama HA, Horsley RD, Steffenson BJ, Schwarz PB, Mesfin A, Franckowiak JD: **Identification of QTLs associated with Fusarium head blight resistance in Zhedar 2 barley**. *Theor Appl Genet* 2003, **108**(1):95-104.
79. de la Pena RC, Smith KP, Capettini F, Muehlbauer GJ, Gallo-Meagher M, Dill-Macky R, Somers DA, Rasmusson DC: **Quantitative trait loci associated with resistance to Fusarium head blight and kernel discoloration in barley**. *Theor Appl Genet* 1999, **99**(3-4):561-569.
80. Mesfin A, Smith KP, Dill-Macky R, Evans CK, Waugh R, Gustus CD, Muehlbauer GJ: **Quantitative trait loci for fusarium head blight resistance in barley detected in a two-rowed by six-rowed population**. *Crop Sci* 2003, **43**(1):307-318.
81. Zhu H, Gilchrist L, Hayes P, Kleinhofs A, Kudrna D, Liu Z, Prom L, Steffenson B, Toojinda T, Vivar H: **Does function follow form? Principal QTLs for Fusarium head blight (FHB) resistance are coincident with QTLs for inflorescence traits and plant height in a doubled-haploid population of barley**. *Theor Appl Gen* 1999, **99**(7-8):1221-1232.
82. Kicherer S, Backes G, Walther U, Jahoor A: **Localising QTLs for leaf rust resistance and agronomic traits in barley (*Hordeum vulgare* L.)**. *Theor Appl Gen* 2000, **100**(6):881-888.
83. Qi X, Niks RE, Stam P, Lindhout P: **Identification of QTLs for partial resistance to leaf rust (*Puccinia hordei*) in barley**. *Theor Appl Gen* 1998, **96**(8):1205-1215.
84. Arru L, Niks RE, Lindhout P, Vale G, Francia E, Pecchioni N: **Genomic regions determining resistance to leaf stripe (*Pyrenophora graminea*) in barley**. *Genome* 2002, **45**(3):460-466.
85. Arru L, Francia E, Pecchioni N: **Isolate-specific QTLs of resistance to leaf stripe (*Pyrenophora graminea*) in the 'Steptoe' x 'Morex' spring barley cross**. *Theor Appl Genet* 2003, **106**(4):668-675.

86. Ma ZQ, Lapitan NLV, Steffenson B: **QTL mapping of net blotch resistance genes in a doubled-haploid population of six-rowed barley**. *Euphytica* 2004, **137**(3):291-296.
87. Manninen O, Kalendar R, Robinson J, Schulman AH: **Application of BARE-1 retrotransposon markers to the mapping of a major resistance gene for net blotch in barley**. *Mol Genet Genomics* 2000, **264**(3):325-334.
88. Raman H, Platz GJ, Chalmers KJ, Raman R, Read BJ, Barr AR, Moody DB: **Mapping of genomic regions associated with net form of net blotch resistance in barley**. *Aust J Agric Res* 2003, **54**(11-12):1359-1367.
89. Gronnerod S, Maroy AG, MacKey J, Tekauz A, Penner GA, Bjornstad A: **Genetic analysis of resistance to barley scald (*Rhynchosporium secalis*) in the Ethiopian line 'Abyssinian' (CI668)**. *Euphytica* 2002, **126**(2):235-250.
90. Bilgic H, Steffenson BJ, Hayes PM: **Molecular mapping of loci conferring resistance to different pathotypes of the spot blotch pathogen in barley**. *Phytopathology* 2006, **96**(7):699-708.
91. Jafary H, Szabo LJ, Niks RE: **Innate nonhost immunity in barley to different heterologous rust fungi is controlled by sets of resistance genes with different and overlapping specificities**. *Mol Plant Microbe Interact* 2006, **19**(11):1270-1279.
92. Castro AJ, Chen XM, Hayes PM, Knapp SJ, Line RF, Toojinda T, Vivar H: **Coincident QTL which determine seedling and adult plant resistance to stripe rust in barley**. *Crop Sci* 2002, **42**(5):1701-1708.
93. Skiba B, Ford R, Pang ECK: **Construction of a linkage map based on a *Lathyrus sativus* backcross population and preliminary investigation of QTLs associated with resistance to ascochyta blight**. *Theor Appl Gen* 2004, **109**(8):1726-1735.
94. Spielmeyer W, Green AG, Bittisnich D, Mendham N, Lagudah ES: **Identification of quantitative trait loci contributing to Fusarium wilt resistance on an AFLP linkage map of flax (*Linum usitatissimum*)**. *Theor Appl Gen* 1998, **97**(4):633-641.
95. Studer B, Boller B, Herrmann D, Bauer E, Posselt UK, Widmer F, Koelliker R: **Genetic mapping reveals a single major QTL for bacterial wilt resistance in Italian ryegrass (*Lolium multiflorum* Lam.)**. *Theor Appl Genet* 2006, **113**(4):661-671.
96. Curley J, Sim SC, Warnke S, Leong S, Barker R, Jung G: **QTL mapping of resistance to gray leaf spot in ryegrass**. *Theor Appl Genet* 2005, **111**(6):1107-1117.
97. Thoquet P, Olivier J, Sperisen C, Rogowsky P, Laterrot H, Grimsley N: **Quantitative trait loci determining resistance to bacterial wilt in tomato cultivar Hawaii7996**. *Mol Plant Microbe Interact* 1996, **9**(9):826-836.
98. Foolad MR, Zhang LP, Khan AA, Nino-Liu D, Lin GY: **Identification of QTLs for early blight (*Alternaria solani*) resistance in tomato using backcross populations of a *Lycopersicon esculentum* x *L-hirsutum* cross**. *Theor Appl Genet* 2002, **104**(6-7):945-958.
99. Bai YL, Huang CC, van der Hulst R, Meijer-Dekens F, Bonnema G, Lindhout P: **QTLs for tomato powdery mildew resistance (*Oidium lycopersici*) in *Lycopersicon parviflorum* G1.1601 co-localize with two qualitative powdery mildew resistance genes**. *Mol Plant Microbe Interact* 2003, **16**(2):169-176.
100. Calenge F, Drouet D, Denance C, Van de Weg WE, Brisset MN, Paulin JP, Durel CE: **Identification of a major QTL together with several minor additive or epistatic

QTLs for resistance to fire blight in apple in two related progenies. *Theor Appl Genet* 2005, **111**(1):128-135.

101. Calenge F, Faure A, Goerre M, Gebhardt C, Van de Weg WE, Parisi L, Durel CE: **Quantitative trait loci (QTL) analysis reveals both broad-spectrum and isolate-specific QTL for scab resistance in an apple progeny challenged with eight isolates of *Venturia inaequalis***. *Phytopathology* 2004, **94**(4):370-379.
102. Liebhard R, Koller B, Patocchi A, Kellerhals M, Pfammatter W, Jermini M, Gessler C: **Mapping quantitative field resistance against apple scab in a 'Fiesta' x 'Discovery' progeny**. *Phytopathology* 2003, **93**(4):493-501.
103. Calenge F, Durel CE: **Both stable and unstable QTLs for resistance to powdery mildew are detected in apple after four years of field assessments**. *Mol Breed* 2006, **17**(4):329-339.
104. Wydra K, Zinsou V, Jorge V, Verdier V: **Identification of pathotypes of *Xanthomonas axonopodis* pv. *manihotis* in Africa and detection of quantitative trait loci and markers for resistance to bacterial blight of cassava**. *Phytopathology* 2004, **94**(10):1084-1093.
105. Musial JM, Aitken KS, Mackie JM, Irwin JAG: **A genetic linkage map in autotetraploid lucerne adapted to northern Australia, and use of the map to identify DNA markers linked to resistance to *Phytophthora medicaginis***. *Aust J Agric Res* 2005, **56**(4):333-344.
106. Nishi T, Tajima T, Noguchi S, Ajisaka H, Negishi H: **Identification of DNA markers of tobacco linked to bacterial wilt resistance**. *Theor Appl Gen* 2003, **106**(4):765-770.
107. Albar L, Lorieux M, Ahmadi N, Rimbault I, Pinel A, Sy AA, Fargette D, Ghesquiere A: **Genetic basis and mapping of the resistance to rice yellow mottle virus. I. QTLs identification and relationship between resistance and plant morphology**. *Theor Appl Genet* 1998, **97**(7):1145-1154.
108. Pressoir G, Albar L, Ahmadi N, Rimbault I, Lorieux M, Fargette D, Ghesquiere A: **Genetic basis and mapping of the resistance to rice yellow mottle virus. II. Evidence of a complementary epistasis between two QTLs**. *Theor Appl Gen* 1998, **97**(7):1155-1161.
109. Tang D, Wu W, Li W, Lu H, Worland AJ: **Mapping of QTLs conferring resistance to bacterial leaf streak in rice**. *Theor Appl Gen* 2000, **101**(1-2):286-291.
110. Li ZK, Arif M, Zhong DB, Fu BY, Xu JL, Domingo-Rey J, Ali J, Vijayakumar CHM, Yu SB, Khush GS: **Complex genetic networks underlying the defensive system of rice (*Oryza sativa* L.) to *Xanthomonas oryzae pv. oryzae***. *Proc Natl Acad Sci U S A* 2006, **103**(21):7994-7999.
111. Miyamoto M, Yano M, Hirasawa H: **Mapping of quantitative trait loci conferring blast field resistance in the Japanese upland rice variety Kahei**. *Breed Sci* 2001, **51**(4):257-261.
112. Sallaud C, Lorieux M, Roumen E, Tharreau D, Berruyer R, Svestasrani P, Garsmeur O, Ghesquiere A, Notteghem JL: **Identification of five new blast resistance genes in the highly blast-resistant rice variety IR64 using a QTL mapping strategy**. *Theor Appl Gen* 2003, **106**(5):794-803.
113. Talukder ZI, Tharreau D, Price AH: **Quantitative trait loci analysis suggests that partial resistance to rice blast is mostly determined by race-specific interactions**. *New Phyt* 2004, **162**(1):197-209.

114. Talukder ZI, McDonald AJS, Price AH: **Loci controlling partial resistance to rice blast do not show marked QTL X environment interaction when plant nitrogen status alters disease severity**. *New Phyt* 2005, **168**(2):455-464.
115. Wu JL, Fan YY, Li DB, Zheng KL, Leung H, Zhuang JY: **Genetic control of rice blast resistance in the durably resistant cultivar Gumei 2 against multiple isolates**. *Theor Appl Gen* 2005, **111**(1):50-56.
116. Tabien RE, Li Z, Paterson AH, Marchetti MA, Stansel JW, Pinson SRM: **Mapping QTLs for field resistance to the rice blast pathogen and evaluating their individual and combined utility in improved varieties**. *Theor Appl Gen* 2002, **105**(2-3):313-324.
117. Li ZK, Pinson SRM, Marchetti MA, Stansel JW, Park WD: **Characterization of Quantitative Trait Loci (Qtls) in Cultivated Rice Contributing to Field-Resistance to Sheath Blight (*Rhizoctonia solani*)**. *Theor Appl Gen* 1995, **91**(2):382-388.
118. Pinson SRM, Capdevielle FM, Oard JH: **Confirming QTLs and finding additional loci conditioning sheath blight resistance in rice using recombinant inbred lines**. *Crop Sci* 2005, **45**(2):503-510.
119. Jones ES, Breese WA, Liu CJ, Singh SD, Shaw DS, Witcombe JR: **Mapping quantitative trait loci for resistance to downy mildew in pearl millet: Field and glasshouse screens detect the same QTL**. *Crop Sci* 2002, **42**(4):1316-1323.
120. Miklas PN, Delorme R, Stone V, Daly MJ, Stavely JR, Steadman JR, Bassett MJ, Beaver JS: **Bacterial, fungal, and viral disease resistance loci mapped in a recombinant inbred common bean population ('Dorado'/XAN 176)**. *J Am Soc Hortic Sci* 2000, **125**(4):476-481.
121. Nodari RO, Tsai SM, Guzman P, Gilbertson RL, Gepts P: **Toward an Integrated Linkage Map of Common Bean .3. Mapping Genetic-Factors Controlling Host-Bacteria Interactions**. *Genetics* 1993, **134**(1):341-350.
122. Tar'an B, Michaels TE, Pauls KP: **Mapping genetic factors affecting the reaction to *Xanthomonas axonopodis* pv. *phaseoli* in *Phaseolus vulgaris* L. under field conditions**. *Genome* 2001, **44**(6):1046-1056.
123. Miklas PN, Johnson WC, Delorme R, Gepts P: **QTL conditioning physiological resistance and avoidance to white mold in dry bean**. *Crop Sci* 2001, **41**(2):309-315.
124. Park SO, Coyne DP, Steadman JR, Skroch PW: **Mapping of QTL for resistance to white mold disease in common bean**. *Crop Sci* 2001, **41**(4):1253-1262.
125. Pilet-Nayel ML, Muehlbauer FJ, McGee RJ, Kraft JM, Baranger A, Coyne CJ: **Quantitative trait loci for partial resistance to Aphanomyces root rot in pea**. *Theor Appl Gen* 2002, **106**(1):28-39.
126. Prioul S, Frankewitz A, Deniot G, Morin G, Baranger A: **Mapping of quantitative trait loci for partial resistance to *Mycosphaerella pinodes* in pea (*Pisum sativum* L.), at the seedling and adult plant stages**. *Theor Appl Gen* 2004, **108**(7):1322-1334.
127. Tar'an B, Warkentin T, Somers DJ, Miranda D, Vandenburg A, Blade S, Woods S, Bing D, Xue A, DeKoeyer D, Penner G: **Quantitative trait loci for lodging resistance, plant height and partial resistance to mycosphaerella blight in field pea (*Pisum sativum* L.)**. *Theor Appl Gen* 2003, **107**(8):1482-1491.

128. Timmerman-Vaughan GM, Frew TJ, Russell AC, Khan T, Butler R, Gilpin M, Murray S, Falloon K: **QTL mapping of partial resistance to field epidemics of ascochyta blight of pea**. *Crop Sci* 2002, **42**(6):2100-2111.

129. Jorge V, Dowkiw A, Faivre-Rampant P, Bastien C: **Genetic architecture of qualitative and quantitative *Melampsora larici-populina* leaf rust resistance in hybrid poplar: genetic mapping and QTL detection**. *New Phyt* 2005, **167**(1):113-127.

130. Decroocq V, Foulongne M, Lambert P, Gall OL, Mantin C, Pascal T, Schurdi-Levraud V, Kervella J: **Analogues of virus resistance genes map to QTLs for resistance to sharka disease in *Prunus davidiana***. *Mol Genet Genomics* 2005, **272**(6):680-689.

131. Foulongne M, Pascal T, Pfeiffer F, Kervella J: **QTLs for powdery mildew resistance in peach x *Prunus davidiana* crosses: consistency across generations and environments**. *Mol Breed* 2003, **12**(1):33-50.

132. Linde M, Hattendorf A, Kaufmann H, Debener T: **Powdery mildew resistance in roses: QTL mapping in different environments using selective genotyping**. *Theor Appl Genet* 2006, **113**(6):1081-1092.

133. Villamon FG, Spooner DM, Orrillo M, Mihovilovich E, Perez W, Bonierbale M: **Late blight resistance linkages in a novel cross of the wild potato species *Solanum paucissectum* (series Piurana)**. *Theor Appl Gen* 2005, **111**(6):1201-1214.

134. Costanzo S, Simko I, Christ BJ, Haynes KG: **QTL analysis of late blight resistance in a diploid potato family of *Solanum phureja* x *S-stenotomum***. *Theor Appl Genet* 2005, **111**(3):609-617.

135. Sorensen KK, Madsen MH, Kirk HG, Madsen DK, Torp AM: **Linkage and quantitative trait locus mapping of foliage late blight resistance in the wild species *Solanum vernei***. *Plant Breed* 2006, **125**(3):268-276.

136. van der Voort JR, van der Vossen E, Bakker E, Overmars H, van Zandroort P, Hutten R, Lankhorst RK, Bakker J: **Two additive QTLs conferring broad-spectrum resistance in potato to *Globodera pallida* are localized on resistance gene clusters**. *Theor Appl Gen* 2000, **101**(7):1122-1130.

137. Klein RR, Rodriguez-Herrera R, Schlueter JA, Klein PE, Yu ZH, Rooney WL: **Identification of genomic regions that affect grain-mould incidence and other traits of agronomic importance in sorghum**. *Theor Appl Gen* 2001, **102**(2-3):307-319.

138. Tao YZ, Jordan DR, Henzell RG, McIntyre CL: **Identification of genomic regions for rust resistance in sorghum**. *Euphytica* 1998, **103**(3):287-292.

139. Brown JS, Schnell RJ, Motamayor JC, Lopes U, Kuhn DN, Borrone JW: **Resistance gene mapping for witches' broom disease in *Theobroma cacao* L. in an F-2 population using SSR markers and candidate genes**. *J Am Soc Hortic Sci* 2005, **130**(3):366-373.

140. Queiroz VT, Guimaraes CT, Anhert D, Schuster I, Daher RT, Pereira MG, Miranda VRM, Loguercio LL, Barros EG, Moreira MA: **Identification of a major QTL in cocoa (*Theobroma cacao* L.) associated with resistance to witches' broom disease**. *Plant Breed* 2003, **122**(3):268-272.

141. Risterucci AM, Paulin D, Ducamp M, N'Goran JAK, Lanaud C: **Identification of QTLs related to cocoa resistance to three species of *Phytophthora***. *Theor Appl Gen* 2003, **108**(1):168-174.

142. Clement D, Risterucci AM, Motamayor JC, N'Goran J, Lanaud C: **Mapping QTL for yield components, vigor, and resistance to *Phytophthora palmivora* in *Theobroma cacao* L**. *Genome* 2003, **46**(2):204-212.

143. Aguilar V, Stamp P, Winzeler M, Winzeler H, Schachermayr G, Keller B, Zanetti S, Messmer MM: **Inheritance of field resistance to *Stagonospora nodorum* leaf and glume blotch and correlations with other morphological traits in hexaploid wheat (*Triticum aestivum* L.)**. *Theor Appl Genet* 2005, **111**(2):325-336.

144. Bariana HS, Hayden MJ, Ahmed NU, Bell JA, Sharp PJ, McIntosh RA: **Mapping of durable adult plant and seedling resistances to stripe rust and stem rust diseases in wheat**. *Aust J Agric Res* 2001, **52**(11-12):1247-1255.

145. Boukhatem N, Baret PV, Mingeot D, Jacquemin JM: **Quantitative trait loci for resistance against Yellow rust in two wheat-derived recombinant inbred line populations**. *Theor Appl Genet* 2002, **104**(1):111-118.

146. Buerstmayr H, Lemmens M, Hartl L, Doldi L, Steiner B, Stierschneider M, Ruckenbauer P: **Molecular mapping of QTLs for Fusarium head blight resistance in spring wheat. I. Resistance to fungal spread (type II resistance)**. *Theor Appl Genet* 2002, **104**(1):84-91.

147. Buerstmayr H, Steiner B, Hartl L, Griesser M, Angerer N, Lengauer D, Miedaner T, Schneider B, Lemmens M: **Molecular mapping of QTLs for Fusarium head blight resistance in spring wheat. II. Resistance to fungal penetration and spread**. *Theor Appl Genet* 2003, **107**(3):503-508.

148. Chantret N, Mingeot D, Sourdille P, Bernard M, Jacquemin JM, Doussinault G: **A major QTL for powdery mildew resistance is stable over time and at two development stages in winter wheat**. *Theor Appl Genet* 2001, **103**(6-7):962-971.

149. Chartrain L, Brading PA, Widdowson JP, Brown JKM: **Partial resistance to septoria tritici blotch (*Mycosphaerella graminicola*) in wheat cultivars arina and riband**. *Phytopathology* 2004, **94**(5):497-504.

150. Christiansen MJ, Feenstra B, Skovgaard IM, Andersen SB: **Genetic analysis of resistance to yellow rust in hexaploid wheat using a mixture model for multiple crosses**. *Theor Appl Genet* 2006, **112**(4):581-591.

151. Eriksen L, Borum F, Jahoor A: **Inheritance and localisation of resistance to *Mycosphaerella graminicola* causing septoria tritici blotch and plant height in the wheat (*Triticum aestivum* L.) genome with DNA markers**. *Theor Appl Genet* 2003, **107**(3):515-527.

152. Faris JD, Friesen TL: **Identification of quantitative trait loci for race-nonspecific resistance to tan spot in wheat**. *Theor Appl Genet* 2005, **111**(2):386-392.

153. Gervais L, Dedryver F, Morlais JY, Bodusseau V, Negre S, Bilous M, Groos C, Trottet M: **Mapping of quantitative trait loci for field resistance to Fusarium head blight in an European winter wheat**. *Theor Appl Genet* 2003, **106**(6):961-970.

154. Jia G, Chen PD, Qin GJ, Bai GH, Wang X, Wang SL, Zhou B, Zhang SH, Liu DJ: **QTLs for Fusarium head blight response in a wheat DH population of Wangshuibai/Alondra's'**. *Euphytica* 2005, **146**(3):183-191.

155. Keller M, Keller B, Schachermayr G, Winzeler M, Schmid JE, Stamp P, Messmer MM: **Quantitative trait loci for resistance against powdery mildew in a segregating wheat x spelt population**. *Theor Appl Gen* 1999, **98**(6-7):903-912.

156. Klahr A, Mohler V, Herz M, Wenzel G, Schwarz G: **Enhanced power of QTL detection for Fusarium head blight resistance in wheat by means of codominant scoring of hemizygous molecular markers**. *Mol Breed* 2004, **13**(4):289-300.
157. Lin F, Kong ZX, Zhu HL, Xue SL, Wu JZ, Tian DG, Wei JB, Zhang CQ, Ma ZQ: **Mapping QTL associated with resistance to Fusarium head blight in the Nanda2419 x Wangshuibai population. I. Type II resistance**. *Theor Appl Gen* 2004, **109**(7):1504-1511.
158. Lin F, Xue SL, Zhang ZZ, Zhang CQ, Kong ZX, Yao GQ, Tian DG, Zhu HL, Li CJ, Cao Y, Wei JB, Luo QY, Ma ZQ: **Mapping QTL associated with resistance to Fusarium head blight in the Nanda2419 x Wangshuibai population. II: Type I resistance**. *Theor Appl Genet* 2006, **112**(3):528-535.
159. Mallard S, Gaudet D, Aldeia A, Abelard C, Besnard AL, Sourdille P, Dedryver F: **Genetic analysis of durable resistance to yellow rust in bread wheat**. *Theor Appl Gen* 2005, **110**(8):1401-1409.
160. Mardi M, Buerstmayr H, Ghareyazie B, Lemmens M, Mohammadi SA, Nolz R, Ruckenbauer P: **QTL analysis of resistance to Fusarium head blight in wheat using a 'Wangshuibai'-derived population**. *Plant Breed* 2005, **124**(4):329-333.
161. Messmer MM, Seyfarth R, Keller M, Schachermayr G, Winzeler M, Zanetti S, Feuillet C, Keller B: **Genetic analysis of durable leaf rust resistance in winter wheat**. *Theor Appl Gen* 2000, **100**(3-4):419-431.
162. Mingeot D, Chantret N, Baret PV, Dekeyser A, Boukhatem N, Sourdille P, Doussinault G, Jacquemin JM: **Mapping QTL involved in adult plant resistance to powdery mildew in the winter wheat line RE714 in two susceptible genetic backgrounds**. *Plant Breed* 2002, **121**(2):133-140.
163. Paillard S, Schnurbusch T, Tiwari R, Messmer M, Winzeler M, Keller B, Schachermayr G: **QTL analysis of resistance to Fusarium head blight in Swiss winter wheat (*Triticum aestivum* L.)**. *Theor Appl Gen* 2004, **109**(2):323-332.
164. Ramburan VP, Pretorius ZA, Louw JH, Boyd LA, Smith PH, Boshoff WHP, Prins R: **A genetic analysis of adult plant resistance to stripe rust in the wheat cultivar Kariega**. *Theor Appl Gen* 2004, **108**(7):1426-1433.
165. Schmolke M, Zimmermann G, Buerstmayr H, Schweizer G, Miedaner T, Korzun V, Ebmeyer E, Hartl L: **Molecular mapping of Fusarium head blight resistance in the winter wheat population Dream/Lynx**. *Theor Appl Gen* 2005, **111**(4):747-756.
166. Schnurbusch T, Paillard S, Fossati D, Messmer M, Schachermayr G, Winzeler M, Keller B: **Detection of QTLs for Stagonospora glume blotch resistance in Swiss winter wheat**. *Theor Appl Gen* 2003, **107**(7):1226-1234.
167. Schnurbusch T, Paillard S, Schori A, Messmer M, Schachermayr G, Winzeler M, Keller B: **Dissection of quantitative and durable leaf rust resistance in Swiss winter wheat reveals a major resistance QTL in the Lr34 chromosomal region**. *Theor Appl Gen* 2004, **108**(3):477-484.
168. Suenaga K, Singh RP, Huerta-Espino J, William HM: **Microsatellite markers for genes Lr34/Yr18 and other quantitative trait loci for leaf rust and stripe rust resistance in bread wheat**. *Phytopathology* 2003, **93**(7):881-890.
169. Yang ZP, Gilbert J, Fedak G, Somers DJ: **Genetic characterization of QTL associated with resistance to Fusarium head blight in a doubled-haploid spring wheat population**. *Genome* 2005, **48**(2):187-196.

170. Zwart RS, Thompson JP, Sheedy JG, Nelson JC: **Mapping quantitative trait loci for resistance to Pratylenchus thornei from synthetic hexaploid wheat in the International Triticeae Mapping Initiative (ITMI) population**. *Aust J Agric Res* 2006, **57**(5):525-530.
171. Avila CM, Satovic Z, Sillero JC, Rubiales D, Moreno MT, Torres AM: **Isolate and organ-specific QTLs for ascochyta blight resistance in faba bean (*Vicia faba* L)**. *Theor Appl Genet* 2004, **108**(6):1071-1078.
172. Roman B, Satovic Z, Avila CM, Rubiales D, Moreno MT, Torres AM: **Locating genes associated with *Ascochyta fabae* resistance in *Vicia faba***. *Aust J Agric Res* 2003, **54**(1):85-90.
173. Chaitieng B, Kaga A, Han OK, Wang XW, Wongkaew S, Laosuwan P, Tomooka N, Vaughan DA: **Mapping a new source of resistance to powdery mildew in mungbean**. *Plant Breed* 2002, **121**(6):521-525.
174. Dintinger J, Verger D, Caiveau S, Risterucci AM, Gilles J, Chiroleu F, Courtois B, Reynaud B, Hamon P: **Genetic mapping of maize stripe disease resistance from the Mascarene source**. *Theor Appl Genet* 2005, **111**(2):347-359.
175. Jones MW, Redinbaugh MG, Anderson RJ, Louie R: **Identification of quantitative trait loci controlling resistance to maize chlorotic dwarf virus**. *Theor Appl Gen* 2004, **110**(1):48-57.
176. Pernet A, Hoisington D, Dintinger J, Jewell D, Jiang C, Khairallah M, Letourmy P, Marchand JL, Glaszmann JC, de Leon DG: **Genetic mapping of maize streak virus resistance from the Mascarene source. II. Resistance in line CIRAD390 and stability across germplasm**. *Theor Appl Gen* 1999, **99**(3-4):540-553.
177. Pernet A, Hoisington D, Franco J, Isnard M, Jewell D, Jiang C, Marchand JL, Reynaud B, Glaszmann JC, de Leon DG: **Genetic mapping of maize streak virus resistance from the Mascarene source. I. Resistance in line D211 and stability against different virus clones**. *Theor Appl Gen* 1999, **99**(3-4):524-539.
178. Welz HG, Schechert A, Pernet A, Pixley KV, Geiger HH: **A gene for resistance to the maize streak virus in the African CIMMYT maize inbred line CML202**. *Mol Breed* 1998, **4**(2):147-154.
179. Xia XC, Melchinger AE, Kuntze L, Lubberstedt T: **Quantitative trait loci mapping of resistance to sugarcane mosaic virus in maize**. *Phytopathology* 1999, **89**(8):660-667.
180. Brown AF, Juvik JA, Pataky JK: **Quantitative trait loci in sweet corn associated with partial resistance to Stewart's wilt, northern corn leaf blight, and common rust**. *Phytopathology* 2001, **91**(3):293-300.
181. Ali ML, Taylor JH, Jie L, Sun G, William M, Kasha KJ, Reid LM, Pauls KP: **Molecular mapping of QTLs for resistance to Gibberella ear rot, in corn, caused by *Fusarium graminearum***. *Genome* 2005, **48**(3):521-533.
182. Carson ML, Stuber CW, Senior ML: **Identification and mapping of quantitative trait loci conditioning resistance to southern leaf blight of maize caused by *Cochliobolus heterostrophus* race O**. *Phytopathology* 2004, **94**(8):862-867.
183. Carson ML, Stuber CW, Senior ML: **Quantitative trait loci conditioning resistance to Phaeosphaeria leaf spot of maize caused by *Phaeosphaeria maydis***. *Plant Dis* 2005, **89**(6):571-574.
184. Clements MJ, Dudley JW, White DG: **Quantitative trait loci associated with resistance to gray leaf spot of corn**. *Phytopathology* 2000, **90**(9):1018-1025.

185. Dingerdissen AL, Geiger HH, Lee M, Schechert A, Welz HG: **Interval mapping of genes for quantitative resistance of maize to *Setosphaeria turcica*, cause of northern leaf blight, in a tropical environment**. *Mol Breed* 1996, **2**(2):143-156.
186. Freymark PJ, Lee M, Woodman WL, Martinson CA: **Quantitative and Qualitative Trait Loci Affecting Host-Plant Response to *Exserohilum turcicum* in Maize (*Zea mays* L)**. *Theor Appl Genet* 1993, **87**(5):537-544.
187. George MLC, Prasanna BM, Rathore RS, Setty TAS, Kasim F, Azrai M, Vasal S, Balla O, Hautea D, Canama A, Regalado E, Vargas M, Khairallah M, Jeffers D, Hoisington D: **Identification of QTLs conferring resistance to downy mildews of maize in Asia**. *Theor Appl Genet* 2003, **107**(3):544-551.
188. Lubberstedt T, Klein D, Melchinger AE: **Comparative quantitative trait loci mapping of partial resistance to *Puccinia sorghi* across four populations of European flint maize**. *Phytopathology* 1998, **88**(12):1324-1329.
189. Lubberstedt T, Klein D, Melchinger AE: **Comparative QTL mapping of resistance to *Ustilago maydis* across four populations of European flint-maize**. *Theor Appl Gen* 1998, **97**(8):1321-1330.
190. Lubberstedt T, Xia XC, Tan G, Liu X, Melchinger AE: **QTL mapping of resistance to *Sporisorium reiliana* in maize**. *Theor Appl Gen* 1999, **99**(3-4):593-598.
191. Nair SK, Prasanna BM, Garg A, Rathore RS, Setty TAS, Singh NN: **Identification and validation of QTLs conferring resistance to sorghum downy mildew (*Peronosclerospora sorghi*) and Rajasthan downy mildew (*P. heteropogoni*) in maize**. *Theor Appl Gen* 2005, **110**(8):1384-1392.
192. Schechert AW, Welz HG, Geiger HH: **QTL for resistance to *Setosphaeria turcica* in tropical African maize**. *Crop Sci* 1999, **39**(2):514-523.
193. Welz HG, Xia XC, Bassetti P, Melchinger AE, Lubberstedt T: **QTLs for resistance to *Setosphaeria turcica* in an early maturing DentxFlint maize population**. *Theor Appl Gen* 1999, **99**(3-4):649-655.
194. Welz HG, Schechert AW, Geiger HH: **Dynamic gene action at QTLs for resistance to *Setosphaeria turcica* in maize**. *Theor Appl Gen* 1999, **98**(6-7):1036-1045.
